# Supplementary figures and images for: Frequent expansion of Plasmodium vivax Duffy Binding Protein in Ethiopia and its epidemiological significance
Source: PLoS Negl Trop Dis. 2019 Sep 11;13(9):e0007222. doi: 10.1371/journal.pntd.0007222 (PMC6756552; doi:10.1371/journal.pntd.0007222)

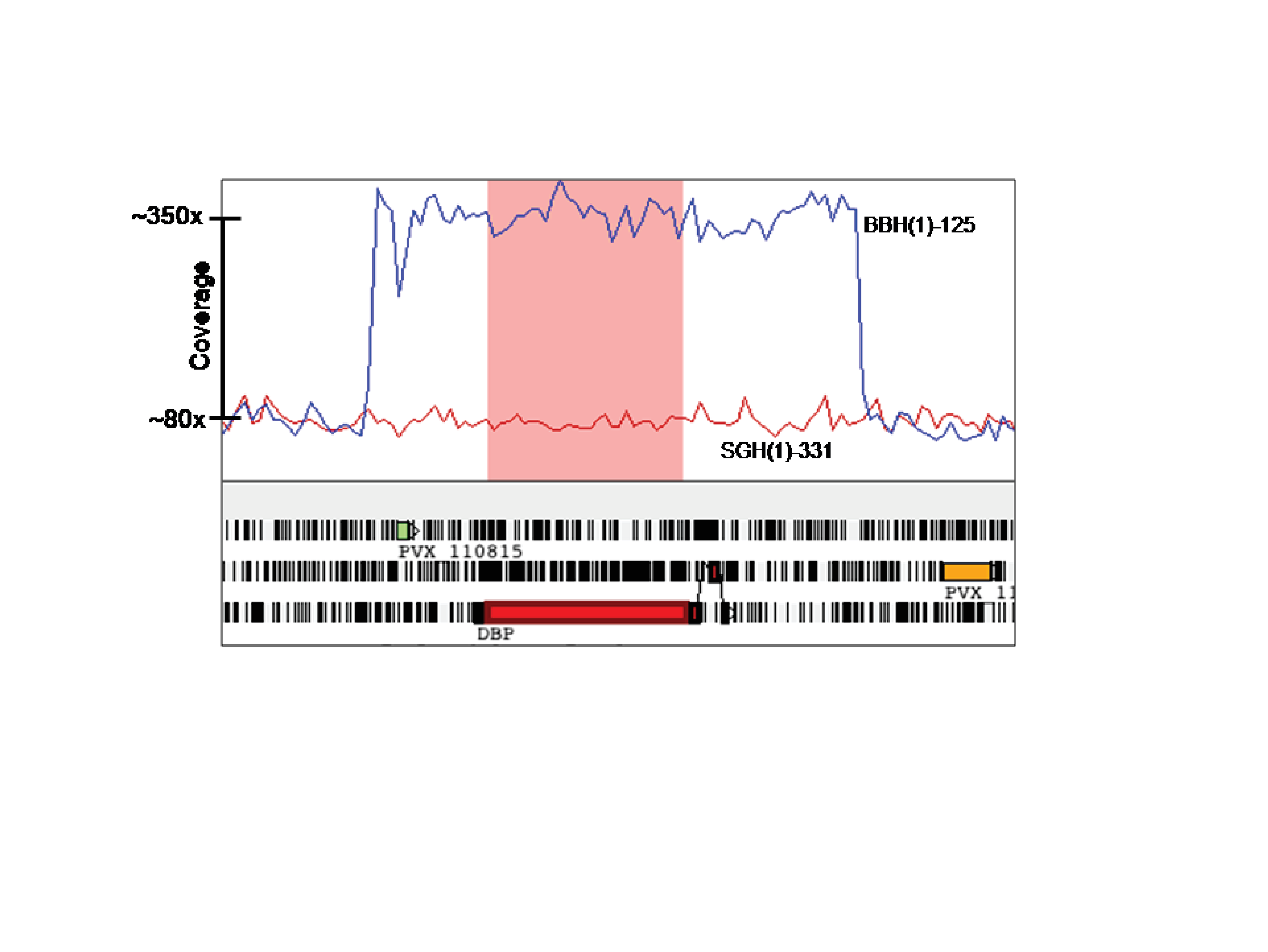

Supplement: S1 Fig — Coverage view showing mapped reads of sample BBH(1)-125 (blue line) with four-fold higher coverage than sample SGH(1)-331 (red line) with respect to P. vivax Sal-1 chromosome 6 region containing PvDBP (red box) using the Artemis genome browser. (TIF) [file pntd.0007222.s007.tif]

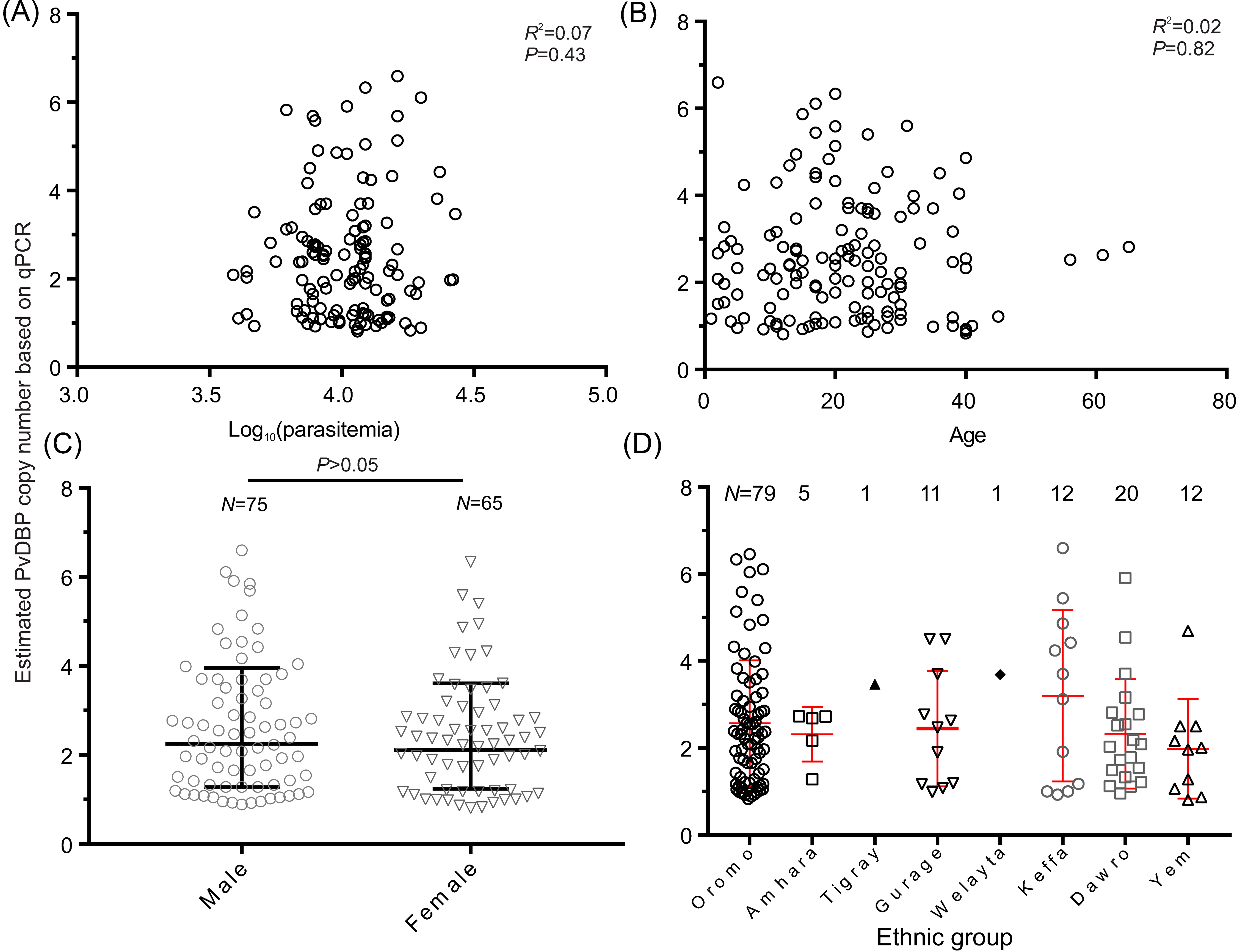

Supplement: S2 Fig — Association plots showing the non-significant correlation of PvDBP gene copy number with (1) parasitemia level, (B) age, (C) gender, and (D) ethnicity of the P. vivax samples. (TIF) [file pntd.0007222.s008.tif]
